# A mobile device-based game prototype for ADHD: development and preliminary feasibility testing

**DOI:** 10.1038/s41398-024-02964-2

**Published:** 2024-06-10

**Authors:** Jie Luo, Fenghua Li, Yuanzhen Wu, Xuanang Liu, Qingyi Zheng, Yanjie Qi, Huanhuan Huang, Gaoyang Xu, Zhengkui Liu, Fan He, Yi Zheng

**Affiliations:** 1 National Clinical Research Center for Mental Disorders, Beijing Key Laboratory of Mental Disorders, Beijing Anding Hospital, Beijing Institute for Brain Disorders Capital Medical University, Beijing, People’s Republic of China; 2 Key Lab of Mental Health, Institute of Psychology, Chinese Academy of Sciences, Beijing, China; 3 Department of Psychology and Human Development, Institute of Education, University College London, London, UK

**Keywords:** ADHD, Human behaviour

## Abstract

This research aimed to devise and assess a mobile game therapy software for children with Attention-Deficit/Hyperactivity Disorder (ADHD), as well as evaluating its suitability and effectiveness in improving the cognitive ability of typically developing children. The study encompassed 55 children diagnosed with ADHD and 55 neurotypical children. Initial assessments involved ADHD-related scales, computerized tests for information processing, and physiological-psychological evaluations. After a 4-week home-based game intervention, participants underwent re-evaluation using baseline measures and provided feedback on treatment satisfaction. Considering the small proportion of study participants who dropped out, data was analyzed using both the Intention-to-Treat (ITT) analysis and the Per-protocol (PP) analysis. The trial was registered at ClinicalTrials.gov (NCT06181747). In ITT analysis, post-intervention analysis using linear mixed models indicated that the ADHD group improved significantly more than the neurotypical group particularly in Continuous Performance Test (CPT) accuracy (*B* = −23.92, *p* < 0.001) and reaction time (*B* = 86.08, *p* < 0.01), along with enhancements in anti-saccade (*B* = −10.65, *p* < 0.05) and delayed-saccade tasks (*B* = 0.34, *p* < 0.05). A reduction in parent-rated SNAP-IV scores was also observed (*B* = 0.43, *p* < 0.01). In PP analysis, paired-sample t-tests suggested that the ADHD group had significant changes pre- and post-intervention, in terms of CPT Accuracy (*t* = −7.62, *p* < 0.01), Anti-saccade task Correct Rate (*t* = −3.90, *p* < 0.01) and SNAP-IV scores (*t* = −4,64, *p* < 0.01). However, no significant changes post-intervention were observed in the neurotypical group. Survey feedback highlighted a strong interest in the games across both groups, though ADHD participants found the game more challenging. Parents of ADHD children reported perceived benefits and a willingness to continue the game therapy, unlike the neurotypical group’s parents. The findings advocated for the integration of serious video games as a complementary tool in ADHD treatment strategies, demonstrating the potential to augment attentional abilities and alleviate clinical symptoms. However, a randomized controlled trial (RCT) is needed to further verify its efficacy.

## Introduction

As one of the most common neuropsychiatric disorders, attention deficit hyperactivity disorder (ADHD) affects 6.4% of Chinese children and adolescents [1]. This disorder is characterized by age-inappropriate inattention, impulsivity and hyperactivity, and is accompanied by cognitive, affective and behavioral deviations. Children with ADHD, accordingly, have their academic performance and emotional development negatively affected. ADHD symptoms have been found to have long-lasting impacts on individuals’ family relationships, partnerships, and overall social functioning [2]. The specific pathogenesis of the disorder is not yet known. It is widely regarded as being influenced by biological, psychological, and social factors – acting synergistically or individually [3]. Most ADHD symptoms persist into adulthood in the absence of treatment, leading to difficulties at home and work [4]. Therefore, early diagnosis and timely intervention are crucial to patients.

Existing treatments for ADHD mainly include medication and non-pharmacological treatments [5]. Specifically, the most popular medications for ADHD are atomoxetine hydrochloride and methylphenidate. Regarding non-pharmacological treatments, behavioral modification, family therapy, psychotherapy, and school education have been proposed as effective. However, the common medications for ADHD have been shown to be ineffective for 18% to 36% of patients [6], and parents have reported psychological side effects as one of the primary reasons for medication discontinuation [7]. Together, these limitations have restricted the applicability of medications, while leading to poor compliance and risks of drug abuse [8]. In addition, as non-pharmacological treatments require high levels of specialized skills, access to appropriate treatment can be limited [9]. Hence, the primary aim of the current research is to develop a treatment that better fits the needs of children with ADHD, improving the effectiveness and applicability of existing treatments.

Recently, research on digital therapies (i.e., software program-driven, evidence-based intervention programs to treat, manage, or prevent disease) has attracted both clinicians’ and researchers’ attention [10]. Video games, as a digital intervention, have been widely applied in facilitating developmental, behavioral, and emotional disorders [11, 12]. Particularly, the engaging virtual cues and fun features of video games satisfy children’s psychological needs, making their uses in therapy appealing to both children and parents [13]. A video game named *Neuro Racer* was found to show a significant effect in promoting participants’ cognitive functioning regarding attention, working memory, and reaction time [14]. Based on this research, Akili Interactive Labs developed a video game (i.e., *EndeavorRx*) targeting the treatment of ADHD in children [15], which has been approved by the U.S. FDA to be marketed as a prescribed treatment. Specifically, this treatment is appropriate for pediatric patients (i.e., aged between 8 and 12 years) with inattention problems [16].

### Cognitive training as a treatment for ADHD

The main component of digital game therapy is cognitive training based on the human brain’s neuroplasticity and its capacity for neural function reorganization [17]. Specifically, these intervention methods aim to improve patients’ executive functions. An increasing number of research has evidenced the positivity of digital games on both cognitive functioning improvements and symptom mitigation. To exemplify, a series of studies have highlighted the effectiveness of cognitive training on inhibitory control [18], working memory [19] planning and time management [20] as well as direct advancements in children with ADHD’s attention performance [21]. However, other similar interventions have failed to replicate previous findings [22, 23]. Taken together, a recent meta-analysis of randomized controlled trials underlined small positive effects of Cogmed on inattention symptoms, however, it did not find significant effects on reducing parent-rated ADHD symptoms or improving academic performance [24]. In addition, training of 20-40 minutes 4-5 days per week, has been proposed to demonstrate optimal effects [25].

However, there are limitations in the previous studies on digital therapy for ADHD. Firstly, despite the increasing high popularity of mobile phones among children, most existing video game therapy software run only on a computer [26]. Without relying on a specific hardware device, mobile serious games demonstrate more portability, thereby higher accessibility and more convenience. Another limitation of existing studies is that they focus solely on cognitive training elements. While cognitive deficits theory partially explains ADHD symptoms, children with ADHD demonstrate more complex clinical manifestations. This is further reflected by the near-zero effect of general Cogmed on far-transfer measures (e.g., cognitive and academic achievement) [26]. The significant individual variability in Cogmed’s efficacy among children with ADHD further highlights the need for improvements in the validity of digital therapies [27]. As many children exhibited low compliance to treatments that involved purely cognitive training, we hypothesized that the lack of a systematic rewards framework and the contextualization of cognitive training are key missing elements in existing cognitive interventions. Particularly, the inclusion of elements such as reward schemes and storylines, which engage user motivation by facilitating a sense of competence and creating a fantasy environment, have been underexplored. Given that ADHD patients often show inconsistencies in self-report motivation and action towards the things of interest and disinterest, we regard treatment engagement as an important puzzle to be solved [28]. As one of the main purposes of scientific gamification is to enhance the motivation factor, we considered a more thorough inclusion of gamification design concepts to be important to video games for treatment purposes.

### Development of the current serious game—“Save the Muse Home”

The fundamental therapeutic goal of the current serious game is to alleviate ADHD symptoms, to reduce social impairment, and thereby improving the prognosis of patients. To improve treatment portability and popularity, we designed the current serious game for mobile and tablet players. The game was designed based on cognitive training principles, rooted in the brain’s capacity to reorganize and adapt through experience [29]. It was also designed with a focus on ADHD symptoms based on the DSM-5 criteria and clinical observations [30]. To explain, the current “Save the Muse Home (SMH)” ADHD therapy game received support from five well-known and experienced clinical practitioners. They provided the practitioner’s perspective on the correspondence between ADHD symptoms and areas of executive function impairment. Meanwhile, we evaluated existing research on Chinese children with ADHD, highlighting patients’ dysfunctions in executive functions including attention, working memory and inhibitory control as the major domains to be targeted [31, 32]. This researcher-practitioner collaboration identified the cognitive impairment elements corresponding to the symptoms, including executive inhibition, motor inhibition, sustained attention, selective attention, working memory, and planning. These areas correspond to cognitive domains trained in previous intervention studies, whereas no known research has attempted training all domains. As a recent meta-analysis of 17 randomized controlled trials proposed that multiple domains of cognitive training, accompanied by moderate training frequency, demonstrate wider clinical benefits [33], the current game design had condensed and integrated different cognitive domains into one specific game.

The training’s effective components were clearly defined and illustrated by children-friendly images in different scenes. The core scenes have been illustrated in Table 1 with corresponding instructions and targeted cognitive domains provided. For example, one scene targeted at enhancing sustained attention and working memory required players to identify the real gems among changing treasure chests. This task demands continuous focused attention and the ability to track multiple targets simultaneously. In addition, we included a planning task through a reward-redemption scheme. Particularly, players were introduced that they could use gems earned in training scenes to exchange for pets (i.e., advanced rewards), provided they saved enough gems to complete the main character’s mission.

**Table 1:** Description of game scene and cognitive domain.

Game Scene	Target Cognitive Domains	Operating Instructions	Picture Examples
Deep sea	Response inhibition	Players keep tapping the sink button to maneuver the warrior to dive through the tunnel, which has lasers firing at irregular intervals and sharks appearing. They need to stop tapping the sink button when sharks appear, while maintaining the player’s attention steady and staying aware of specific missions as they appear.	
	Interference inhibition		
	Sustained attention		
Lava statue	Working memory	Players follow the red path that changes to reach the stone statue as required, with green paths interfering during the process.	
Olympic torch	Reaction	Players summon the flame power to defeat the demon by clicking on the labeled balls in sequence, the position of which is constantly changing. Players train the reaction speed by tracking and capturing the target, and quickly finding the target requires steady attention.	
	Selective attention		
Desert ruins	Sustained attention	Players find the real gems in the changing treasure chests. Players need to focus on the stability of attention during the changes and keep steady track of multiple targets.	
	Working memory		
Psychedelic forest	Cognitive shift	Players defeat the demonic monsters with the collection of correct elements. Players are trained to perform good task transitions and focused attention.	
	Cognitive flexibility		

Moreover, we specifically focused on enhancing players’ experiences and engagement by refining the gamification elements such as the reward mechanism, storyline, aesthetics, etc. To elaborate, the game’s reward mechanism was designed to increase players’ motivation through both fixed and variable ratio reward schemes. We also maintained a moderate challenge level across game levels to engage players effectively. Furthermore, to the best of our knowledge, the current game is one of the first ADHD digital treatment attempts to weave various training scenes into a comprehensive storyline. Specifically, the storyline features an empathetic character with successive narratives, responsiveness to players, and a fantasy theme, depicting the story of a warrior striving to save his homeland.

After completing the design of the serious game (i.e., Save the Muse Home), we conducted this study for the preliminary validation of its therapeutic effectiveness. To ensure the objectivity and accuracy of the assessment results, this study adopted a combination of well-established methods from three aspects: questionnaire measurements, computer-assisted information processing tests, and psychobiological assessments. Based on previous studies, it is hypothesized that participants’ attention function would be better after the 4-week intervention. However, as participants in previous studies were generally ADHD children, we also wanted to investigate whether this cognitive intervention would improve the cognitive ability of neurotypical children. Therefore, in this study, we recruited typically developing children to investigate whether the same direction of attentional function change that occurs in ADHD children after training can be replicated with children who demonstrate different cognitive levels. As a feasibility study, it is possible to identify the population that would benefit from this game. Since this game design was specifically tailored to address ADHD symptoms, we anticipate that although both the ADHD and the neurotypical group participants would improve, children in the ADHD group will demonstrate greater progress compared to those in the neurotypical group, given the potentially greater room for improvement among individuals with ADHD.

## Methods and materials

### Participants

The study included 55 ADHD patients and 55 typically developing participants, with ADHD participants being outpatients of Beijing Anding Hospital. Inclusion criteria for children were: (a) aged 6–12 years; (b) meeting DSM-5 diagnostic criteria for ADHD; (c) were newly diagnosed cases or had been off medication for at least two weeks. Both patients/guardians and participants aged 8 and above provided informed consent. Exclusion criteria included: (a) neurodevelopmental disorders like cerebral palsy; (b) severe mental illnesses such as Tourette’s disorder or autism; (c) auditory disorders mimicking ADHD; (d) use of psychotropic drugs during the study; (e) ineffectiveness after two ADHD medication trials; (f) inability to use software, e.g., color blindness; (g) past or current gaming addiction. This exclusion criteria was adopted to select a homogeneous sample. The neurotypical group was recruited from a middle school in Nanjing, China, matched by age and sex with the ADHD group. The study adhered to the Declaration of Helsinki and was approved by the Ethics Committee, Institute of Psychology, Chinese Academy of Sciences (H23067), with all participants volunteering for the study. This trail is registered at ClinicalTrials.gov: NCT06181747.

### Performance measures

#### Swanson Nolan, and Pelham-IV rating scales (SNAP-IV)

The SNAP-IV rating scale [34] is a revised version of the Swanson, Nolan, and Pelham (SNAP) scale, developed based on the ADHD symptom descriptions in the Diagnostic and Statistical Manual of Mental Disorders (DSM). It comprises three subscales: attention deficit, hyperactivity-impulsivity, and oppositional defiance. Items are rated on a scale from 0 to 3, with the mean value of each subscale being calculated. It is primarily used for screening, diagnostic support, and assessing treatment efficacy and symptom improvement in children and adolescents aged 6 to 18 years. The reliability ranges from 0.68 to 0.84.

#### Strengths and difficulties questionnaire (SDQ)

The SDQ is a brief behavioral screening questionnaire created by American psychologist Goodman R. in 1997 based on DSM-IV and ICD-10 [35]. This tool assesses behavioral and emotional problems in children and adolescents, offering a reliability range of 0.84 to 0.96.

#### Behavior rating inventory of executive function (BRIEF)

The BRIEF rating scale, developed by Gioia et al., assesses executive functioning in children aged 6 to 18 years, both at home and in school settings [36]. In this study, parents completed the scale based on their observations of their children. The scale features 86 items divided into two main areas: the Behavior Management Index (BMI), encompassing the Inhibition, Conversion and Affect, and Control subscales; and the Metacognitive Functioning Index (MFI), which includes five subscales: Task Initiation, Working Memory, Planning, Organization, and Monitoring. High scores indicate more severe functional impairment. The reliability ranges from 0.68 to 0.96.

### Neurocognitive/attentional task

#### Continuous performance test (CPT)

The CPT software is one of the most commonly used experimental paradigms for examining attention. It is a computer-assisted information processing test that provides objective measures independent of supervisors [37]. The results of the CPT reflect the participant’s ability to sustain attention and to inhibit and control impulses. The classic CPT paradigm involves rapidly presenting a series of stimuli (i.e., numbers or characters) on a computerized monitor, requiring participants to respond to a pre-specified target. In this study, the CPT paradigm included a white boxed area on a black background on the calculator screen, with white squares randomly appearing above or below the boxed area. Participants were required to press the right mouse button when a square appeared below the boxed area. Children were instructed to click the mouse for “go” stimuli and to refrain from clicking for “no-go” stimuli. Each stimulus was presented for 500 ms, with a 1000 ms interval between every two trials. All children completed two sets of tasks, each consisting of 252 trials. The first set involved 56 “go” and 196 “no-go” trials, while the second set comprised 196 “go” and 56 “no-go” trials.

According to the signal detection theory, indices relatively unaffected by other response criteria can be calculated (i.e., d’). d’ is a sensitivity index in signal detection tasks that reflects the participant’s perceptual sensitivity, evaluating the accuracy and discrimination ability in detecting signal [38]. A higher d’ value indicates accurate signals and the ability to distinguish between target and non-target stimuli. Thus, the main measures of the CPT are reaction time, accuracy (i.e., the number of correct responses to the target stimulus), commission error (i.e., responses to non-target stimuli, a measure of impulsivity), d’ (i.e., caculated as Z_accuracy_ - Z_commission error_).

#### Eye movement analysis

Eye movement tasks are important psychological measures that have been widely adopted and developed based on the strong correlation between attention and eye movement [39]. The degree to which individuals focus on or divert from a task has been evidenced to influence their eye movement trajectory, underlining eye movement as an external indicator of attention. Different eye movement tasks reflect different cognitive behaviors [40]. In this study, we used anti-saccade and delayed-saccade tasks to examine participants’ cognitive functions, including inhibition and working memory [41, 42]. The task descriptions are as follows:

##### Anti-saccade task

Initially, the central fixation point cross is presented for 2 seconds. Afterward, as the central cross disappears, a target green dot randomly appears at four positions (i.e., 3°, 6° to the left and right sides horizontally) [41]. Each target dot is presented at each position for 1000 ms, five times, totaling 20 trials. Following the response time, a 300-ms feedback point appears in the correct position. To complete the anti-saccades task, participants must [38]: a) inhibit the impulse to look at the suddenly appearing stimulus; b) calculate the spatial position opposite and equidistant from the stimulus; and c) initiate volitional saccades towards the point where no stimulus appears.

##### Delayed-saccade task

Similarly, the central fixation point cross was also presented for 2 s in this task. As the cross remains on the screen, a target green dot stimulus appears randomly at four positions (i.e., 3° and 6° to the left and right sides horizontally) for 100 ms [42]. This is followed by the gaze point being presented only at the center of the screen for 1, 3, and 5 s, followed by a blank screen for 1.4 s. Participants were required to focus on the cross while the green dots were presented, and to recall the original position of the target dots when the blank screen appeared. Finally, a 200-ms feedback point appeared at the correct location. In the delayed-saccade tasks, participants were required to concentrate on the central fixation point, and make a saccade only when this point disappeared. The number of early saccades (i.e., trials in which the participants did not wait until the disappearance of the central gaze point to perform a saccade) in the delay saccade task reflected the participants’ inhibitory control. Participants were also required to recall the location of the stimulus point before making a saccade. This measure primarily reflects their spatial working memory.

In saccade tasks, the actual saccade refers to the observed eye movement when shifting gaze from one point to another. The target saccade represents the intended or planned eye movement, based on the instructions or requirements of a task or experimental paradigm. In the anti-saccade task, participants were required not only to make a saccade in the opposite direction of the stimulus but also to estimate an equal distance in the opposite direction of the stimulus for working memory assessment. The difference between the actual and the target saccade is calculated to represent the participant’s working memory, with a small difference indicating high working memory capacity.

### Safety and satisfaction survey

Although participants had been informed about potential safety issues they might experience and were assured about their right to withdraw at any time during the study, we conducted a safety survey to further evaluate safety concerns. Specifically, this survey was conducted with parents, who were instructed to report any adverse incidents to the researchers via phone during this intervention study. After the study’s completion, all child participants and their parents were verbally interviewed to gather their overall impression of the game therapy and specific cognitive training tasks. The data were recorded and analyzed by researchers.

### Procedure

ADHD patients were recruited through information posted at the outpatient clinic of Anding Hospital. The ADHD patient group consisted of individuals with a clinician-confirmed diagnosis of ADHD who met the study’s inclusion and exclusion criteria. Participants were either not on medication or had stopped taking medication for at least two weeks before enrollment, after assessing the safety of discontinuing medication during the intervention period. Informed consent was obtained from the participants and the family members. Neurotypical participants were enrolled after screening confirmed that they were normally developing children. All participants underwent baseline measures using scales, CPT tests, and eye movement tasks before the intervention.

Following a brief intermission, researchers introduced the game mechanics and the training method to the children and their families. The children then engaged in 20-minute primary training sessions, during which any discomfort the children experienced was recorded. Drawing on previous findings, the intervention period lasted four weeks, with participants being instructed to engage in the game training at home for five days per week [18]. Consistent with literature proposing that treatments of at least 20 minutes are more effective, this intervention study prescribed 20-25 min of training per day [25]. To prevent game addiction, a maximum engagement time of 30 minutes per day was set. Participants were encouraged to undertake game training as per their capability. An adaptive algorithm was implemented to adjust the difficulty of the game, ensuring a moderate challenge for the player and informing them about level progression. In each game scene, participants automatically advanced to the next difficulty level upon breaking their own records for three times or achieving the set goals for each level. Participants were followed up weekly to ensure compliance rate, and the training duration was tracked. The final assessment was conducted after the 28th day, and the measures were consistent with the baseline measures conducted during the pre-intervention period.

### Data analysis

Data analysis was stratified into two components: the Intention-to-Treat (ITT) analysis and the Per-protocol (PP) analysis. These approaches aimed to comprehensively evaluate the impact of a four-week electronic game-based intervention on individuals with Attention-Deficit/Hyperactivity Disorder (ADHD) compared to a neurotypical group. Firstly, descriptive statistics on the general demographic information of the ADHD and neurotypical groups were analyzed, categorical variables were summarized with frequencies and percentages, and continuous variables were described using means and standard deviations. For ITT analysis, a linear mixed model was employed. This model considered each evaluation measure result as a dependent variable such as the CPT, and SNAP-IV, SDQ, BRIEF and eye movement tasks, with group, time, and the interaction between group and time as fixed effects. The model accounted for repeated measures within participants. False Discovery Rate (FDR) (Benjamini & Hochberg, 1995) was applied to correct results for multiple comparisons. For PP analysis, Paired t-tests were utilized for this excluding drop-out date, comparing cognitive-behavioral outcomes before and after the intervention within each group. Bonferroni correction was applied to counteract the potential issue of multiple comparisons. The findings are considered statistically significant when adjusted p < 0.05. All analyses were performed in the open-source statistical package R (v.3.3.1). Only ITT analyses are reported in the result section of this report, and the PP analyses may be found in the supplementary materials.

## Results

### Participant flow

**Fig. 1:**
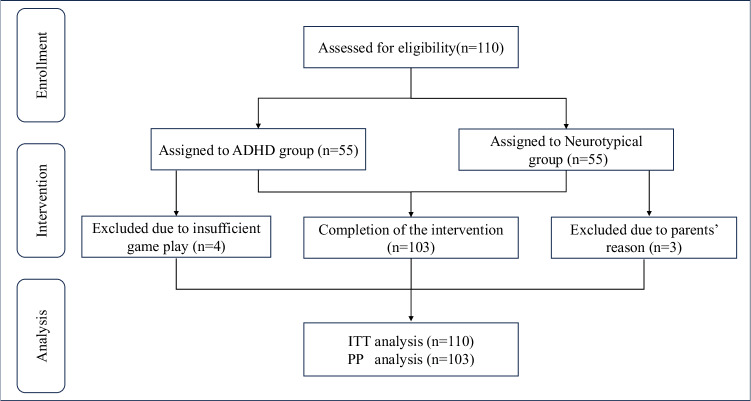
CONSORT flow diagram. ITT intention-to-treat, PP per-protocol.

A total of 55 ADHD and 55 neurotypical participants were recruited in the study. Based on the backstage data requirements of the minimum weekly cumulative training time of 100 min (i.e., 20 min per day for 5 days), four ADHD patients were excluded from the study’s PP analyses. Also, three neurotypical participants withdrew from the study due to their families’ unwillingness to complete the game training. All participants had at least one training record as well as the baseline test data being recorded, thus all participants were included in the ITT analysis. This left 51 ADHD participants and 52 neurotypical participants in the PP analysis of the study, and the data were available for inclusion in the statistics based on the enrollment and participation process illustrated in Fig. 1.

### Demographic information

In the ITT analysis, the 55 participants (male = 41; female = 14) in the ADHD group had a mean age of 8.53 ± 1.32 years, and the average age of the 55 participants (male = 43; female= 12) in the neurotypical group was 8.87 ± 1.68 years old. 18 (32.73%) participants in the ADHD group had previous past medication uptake experiences. In the PP analysis, the 51 participants (male = 39; female = 12) in the ADHD group included had a mean age of 8.39 ± 1.25 years, Prior stimulant use 16 (31.37%), and the average age of the 52 participants (male = 40) in the neurotypical group was 8.64 ± 1.55 years old. The age and gender differences between the two groups were not significant (*p* > 0.05) in both the ITT and the PP analysis, and were comparable.

### Group differences at baseline (Time 1)

As reported in Table 2, before the initiation of the digital game-based intervention (Time 1), significant group differences were observed in various cognitive and behavioral measures. To elaborate, the ADHD group exhibited significantly lower accuracy in the Continuous Performance Test (CPT) compared to the neurotypical group (*B* = 7.80, *p* < 0.01). Behavioral performances as measured by the Behavioral Regulation Index (*B* = 13.65, *p* < 0.001) and the Metacognition Index (*B* = 21.26, *p* < 0.001) of the Behavior Rating Inventory of Executive Function (BRIEF), were also significantly higher in the ADHD group. The SNAP-IV scale indicated significantly higher inattention (*B* = 0.79, *p* < 0.001) and hyperactivity-impulsivity (*B* = 0.65, *p* < 0.001) scores in the ADHD group.

**Table 2:** The Linear mixed models for each CPT, Saccade task, and Rating scales metric.

	Group	Time	ADHD × Time	Constant
*CPT*				
Accuracy	7.80^**^ (2.67)	0.77 (1.75)	−23.92^***^ (3.28)	68.22 (1.22)
Commission error	−6.80^**^ (2.03)	**−**0.46 (1.56)	15.47^***^ (2.81)	26.30 (1.13)
Reaction time	−62.47^**^ (21.87)	**−**12.27 (17.67)	86.08^**^ (29.63)	539.10 (12.72)
D-prime,d'	0.66^***^ (0.17)	0.04 (0.10)	−1.34^***^ (0.20)	1.16 (0.07)
*Saccade task*				
Correct rate in Anti-saccade task	5.10 (3.50)	2.26 (3.36)	−10.65^*^ (4.83)	20.19 (2.18)
Accuracy in the Anti-saccade Task	−0.05^**^ (0.02)	0.01 (0.02)	0.12^*^ (0.06)	0.13 (0.02)
Number of early saccades in Delay-saccade task	**−**0.14 (0.10)	**−**0.07 (0.10)	0.34^*^ (0.16)	0.82 (0.07)
Accuracy in the Delay-saccade Task	0.03 (0.02)	0.01 (0.01)	0.02 (0.02)	0.06 (0.01)
Rating scales				
*SNAP-IV*				
Inattention	0.79^***^ (0.11)	**−**0.18 (0.10)	0.51^**^ (0.15)	0.95 (0.08)
Hyperactivity-Impulsivity	0.65^***^ (0.14)	**−**0.16 (0.11)	0.57^**^ (0.18)	0.69 (0.09)
Total score	0.71^***^ (0.11)	**−**0.14 (0.09)	0.43^**^ (0.14)	0.81 (0.08)
SDQ	6.70^***^ (1.16)	**−**0.43 (1.19)	0.67 (1.73)	12.48 (0.76)
*BRIEF*				
Behavioral Regulation Index, BRI	13.65^***^ (2.31)	0.04 (2.09)	4.60 (3.27)	51.29 (1.53)
Metacognition Index, MI	21.26^***^ (3.52)	**−**1.48 (3.44)	11.05^*^ (5.02)	80.12 (2.46)

Values indicate the estimated effect and corresponding standard error (SE). a: The parameter was set to zero in the mixed-effects model due to redundancy.

*CPT* Continuous Performance Test, *SDQ* Strengths and Difficulties Questionnaire, *BRIEF* Behavior Rating Inventory of Executive Function.

*Adjusted *p* < 0.05.**Adjusted *p* < 0.01. ***Adjusted *p* < 0.001. Bold indicates significance after FDR correction.

### Intervention effects (Group × Time Interaction)

**Fig. 2:**
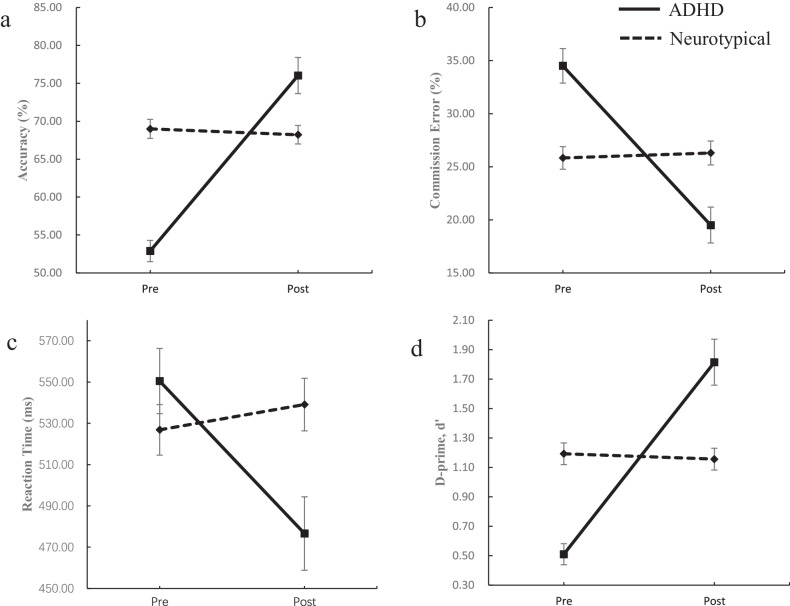
Estimated marginal means (y-axis) for performance on the CPT in the ADHD and neurotypical groups across the pre- and post-intervention time points (x-axis). Error bars indicate standard E for the ADHD and Neurotypical groups. **a** Accuracy, **b** commission error, **c** reaction time, **d** D-prime, d’.

**Fig. 3:**
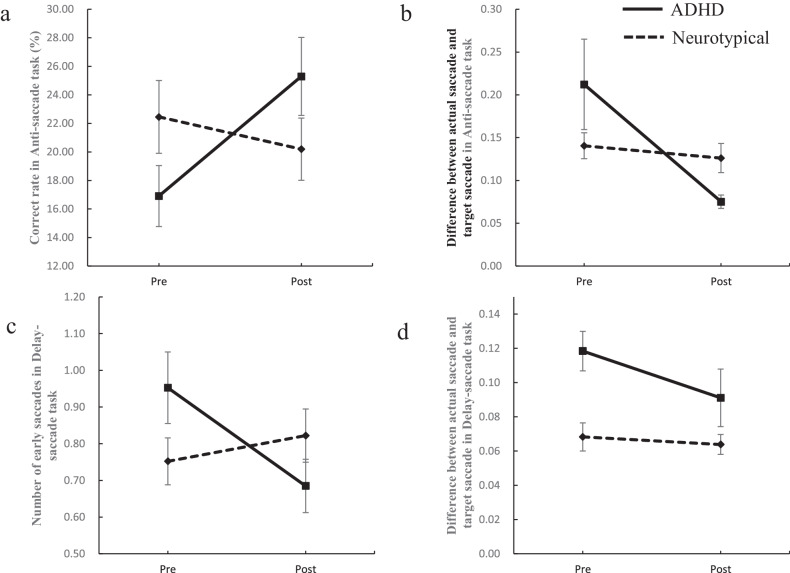
Estimated marginal means (*y*-axis) for performance on the saccade task between the ADHD and Neurotypical groups across the pre-and post-intervention time points (*x*-axis). Error bars indicate standard E for the ADHD and Neurotypical groups. **a** Correct rate in Anti-saccade task. **b** Difference between actual saccade and target saccade in the Anti-saccade task. **c** Number of early saccades in Delay-saccade task. **d** Difference between actual saccade and target saccade in Delay-saccade task.

**Fig. 4:**
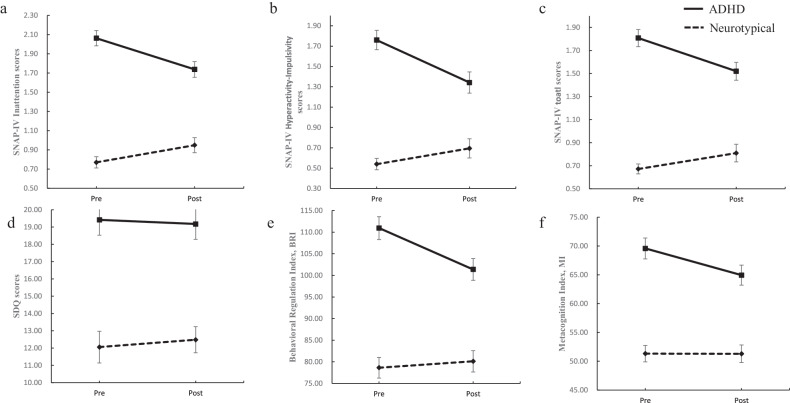
Estimated marginal means (*y*-axis) for performance on the scales between the ADHD and Neurotypical groups across the pre-and post-intervention time points (*x*-axis). Error bars indicate standard E for the ADHD and Neurotypical groups. **a**–**c** SNAP-IV scales, **d** SDQ scales, **e**, **f** BRIEF scales.

The impact of the four-week game-based intervention was assessed by examining the Group × Time interaction effects (Table 2). Figures 2, 3, 4 show the performance on the different evaluations with corresponding standard errors (SE) across time for each group. Regarding CPT, which is the primary outcome of this research [13], differences between the pre-and post-intervention trends of the ADHD group and the neurotypical group were observed. Children in the ADHD group showed a significant change in most of the indicators after the intervention, whereas those in the neurotypical group showed little change before and after the intervention. the ADHD group demonstrated a significant improvement in accuracy post-intervention compared to the neurotypical group (*B* = -23.92, p < 0.001), Reaction time significantly decreased in the ADHD group post-intervention, suggesting enhanced cognitive processing speed (*B* = 86.08, *p* < 0.01). Also, D-prime (d’) showed a significant increase in the ADHD group, indicating improved discrimination ability (*B* = -1.34, *p* < 0.001).

Regarding the secondary outcome, in terms of the saccade task, the cognitive performances measured by both the correct rate in the Anti-saccade Task (*B* = -10.65, *p* < 0.05) and the number of early saccades in the Delayed-saccade Task (*B* = 0.34, *p* < 0.05), improved significantly in the ADHD group post-intervention compared to the neurotypical group. Moreover, significant reductions in SNAP-IV scores post-intervention were observed in the ADHD group for inattention (*B* = 0.51, *p* < 0.01), hyperactivity-impulsivity (*B* = 0.57, *p* < 0.01), and total symptoms (*B* = 0.43, *p* < 0.01). In addition, scores on the Metacognition Index significantly improved in the ADHD group post-intervention compared to the neurotypical group (*B* = 11.05, *p* < 0.05), whereas no statistically significant effect on the Behavioural Regulation Index (*B* = 4.60, *p* > 0.05) or the Strengths and Difficulties Questionnaire (SDQ) in the ADHD group was observed compared to the neurotypical group (*B* = 0.67, *p* > 0.05). Furthermore, the results in the PP analyses were similar, with ADHD showing a significant improvement in attentional functioning pre-and post-intervention. Significant positive changes in various indicators such as CPT Accuracy (*t* = −7.62, *p* < 0.01), Anti-saccade task Correct Rate (*t* = −3.90, *p* < 0.01) and SNAP-IV scores (*t* = −4,64, *p* < 0.01) were also observed (see Supplementary Table 1). Conversely, no significant intervention effect on the neurotypical group had been observed, despite the Bonferroni-corrected *t* tests revealed some improvements in accuracy in the Anti-saccade task (*t* = 2.32, *p* < 0.05) and scores on SNAP-IV (*t* = −2.10, *p* < 0.05) post-intervention (see Supplementary Table 2).

### Adverse effects and satisfaction survey

A total of three adverse reactions were reported during this study. Two were from the neurotypical group (i.e., dizziness #1009 and vomiting #1034) and one was from the ADHD group (i.e., dizziness #1002). These reactions were evaluated by the PI and the researchers to be unrelated to the game. At the end of the study, parents and children were surveyed about their satisfaction with the game software. All participants in the study completed the interview, and the final results revealed that the children participants were satisfied with the game, and found it to be more fun. However, in the survey on the difficulty of the game, it was found that the majority of the children participants in the neurotypical group found the game easy, while the children in the ADHD group found it difficult to complete the game. Parents in the parent survey were more supportive of the game therapy in the ADHD group, and more parents were willing to continue the video game training after the study was over. However, a greater number of parents in the neurotypical group reported that the game was relatively simple and expressed concerns that it might negatively affect their children’s learning by consuming valuable time. They also showed more resistance to video game training, as shown in Table 3.

**Table 3:** Parent and child feedback on SMH after the intervention.

	ADHD (n = 51)	Neurotypical (n = 52)
*Child Questions*		
“How interesting was SMH to play?” (1 = boring, 10 = fun), mean ± SD	7.92 ± 0.69	6.02 ± 0.58
“How challenging was SMH to play?” (1 = Easy, 10 = Hard), mean ± SD	7.69 ± 0.92	4.80 ± 0.91
*Parent Questions*		
“Did you find it easy to understand the game?” (1 = Easy, 10 = Hard), mean ± SD	8.10 ± 0.90	8.59 ± 0.78
“Do you think it was useful for your child to spend time playing SMH?” (1 = useless, 10 = very useful), mean ± SD	8.18 ± 0.97	4.72 ± 0.91
“Would you like your child to continue playing SMH after this program?”		
Yes, *n* (%)	38 (74.50%)	11 (21.15%)
No, *n* (%)	4 (7.84%)	22 (42.31%)
Not sure, n (%)	9 (17.65%)	19 (36.54%)

## Discussion

The main aim of this study was to investigate the efficacy of a self-developed serious game software on treating children with ADHD over 4 weeks. A key component of the concept validation study was selecting established methods that accurately depicted the functional impairments characteristic of ADHD patients, aiming to evaluate clinically relevant outcomes. To ensure the objectivity and accuracy of the assessment results, this study adopted a combination of well-established methods from three aspects: questionnaire measurements, computer-assisted information processing tests, and psychobiological assessments.

The preliminary results confirmed that the serious game software effectively improved attention, inhibitory control, planning, and working memory in ADHD patients. The widely-used SNAP-IV and BRIEF scales were adopted to evaluate the symptoms and functional impacts of ADHD. After the 4-week intervention, significant reductions in inattention and hyperactive-impulsiveness as measured by SNAP-IV were observed, demonstrating the game’s significant benefits in improving ADHD clinical symptoms. Significant reductions in both the Behavioral Regulation Index and the Metacognition Index on the BRIEF scale were also noted for the ADHD group pre- and post-intervention, indicating reduced cognitive impairment and underscoring the effectiveness of the game training. However, no significant differences were observed on the Strengths and Difficulties Questionnaire (SDQ), which assesses children’s psychological health and behavioral problems [35].

This may be related to limitations in the study’s use of the rating scales, as questionnaire measures can be influenced by the subjective judgments of the participants [43]. Given that the current study was an open-label, developmental, and feasibility trial, the improvements reported by parents might be subject to their impressions of the intervention. For instance, parents of ADHD patients may possess more knowledge about ADHD than parents of children in the control group, potentially increasing the sensitivity during the questionnaire evaluation process. Moreover, parents’ varying attitudes towards gaming could influence their assessments before and after the intervention. Consequently, questionnaire assessments were utilized only as one component of the efficacy evaluation in this study. To ensure objectivity, we also employed computer-assisted information processing tests and psychobiological assessments.

Among these, the CPT, a widely used tool for assessing continuous attention, was also adopted in this study to provide objective results. The CPT metrics include hits, misses, false alarms, and correct rejections. Misses are indicative of participants’ attention deficit, while false alarms reflect impulsivity [38]. In this study, we observed that ADHD patients exhibited high rates of misses and false alarms before the intervention. However, both rates significantly decreased post-intervention, suggesting that game training positively affects ADHD patients’ sustained attention and inhibitory control. Additionally, the d’ value (i.e., the perceptual sensitivity index) of the ADHD group significantly increased post-intervention.

It is important to note that since the game training’s underlying mechanism is cognitive training, there may be some overlap with the content assessed, potentially influencing the validity of evaluation results. This overlap could lead to misinterpretation by doctors and parents regarding a child’s actual abilities [44]. To address this, we highlighted distinctions between the game software and the CPT software evaluation in this study, including 1) the game software featuring both single-task and multi-task conditions, in contrast to CPT’s single-task conditions; 2) changing rule conditions in the game software with game progression, unlike the static rules in the CPT; and 3) the game tasks being set in a rich virtual environment, whereas CPT tasks are presented with monochromatic and geometric stimuli. Given these differences, we argue that CPT results can indeed represent improvements in ADHD patients’ attentional abilities. Additionally, this study incorporated eye movement tests, assessing attention from a biopsychological perspective, independent of the game’s perceptual demands. The ADHD group showed significant improvements in the anti-saccade and delayed-saccade task post-intervention, which gauge inhibitory control and working memory, respectively – key areas of cognitive impairments in ADHD [45–48]. Improvement in eye movement performance indirectly highlights improvement in the main physiological domains responsible for volitional eye-tracking, such as the frontal-striatal loop [49], aligning with the game training’s therapeutic principle of neuroplasticity. Therefore, eye movement testing not only assesses changes in executive function pre- and post-intervention but also offers biological evidence for the training’s effectiveness [50].

It is worth mentioning that in this study, the game intervention had a larger effect on improving participants’ scores on cognitive tasks. A smaller yet statistically significant effect was observed in their performance as measured by rating scales. One potential explanation is that cognitive training aimed at improving executive function may have limited transfer effects on symptom relief [26], despite children complying with the treatment. Consequently, the transfer of effects from trained cognitive domains to general cognitive abilities might have experienced a degree of loss. Nevertheless, the improvements observed in the ADHD group were definitive and reliable, with participants from both groups providing positive feedback on the game’s playability, underlining its effectiveness. Additionally, the sample size in the current research was sufficient to demonstrate the positive effects of the current game intervention in reducing ADHD symptoms. Therefore, it holds significance for future research on digital therapy for ADHD to continue exploring methods to enhance the transfer of effects.

It is also important to explore the reasons behind the lack of improvements in the cognitive performance of the neurotypical group, as measured by the scales before and after the intervention. For this group, only the accuracy in the anti-saccade task showed significant improvement. One possible explanation might be that the neurotypical participants already had higher baseline levels of cognitive functioning, leaving less room for further enhancement. The study revealed that the neurotypical group completed the maximum difficulty levels in significantly less time than the ADHD group (ADHD : neurotypical = 26.41 days : 17.37 days), suggesting that the training effect may be diminished for neurotypical participants in the latter half of the training. This implies that the game might not have been challenging enough for the control group, potentially leading to reduced engagement [51]. Furthermore, parental attitudes toward play therapy could influence participant engagement during the training process and, consequently, the training effectiveness [52]. At the end of the program, we also gathered feedback on the game from the children and their parents, finding that families in the ADHD group had more positive attitudes towards game therapy and were more willing to have their children participate in game training. In contrast, parents in the control group expressed a more negative attitude toward the games, consistent with previous studies [53], which might have contributed to the absence of significant changes in the neurotypical group pre-and post-intervention. The minor changes in the performance of the neurotypical group suggest that the game’s difficulty setting may need to be better tailored to the participants. In future iterations of the game, we plan to adjust the game’s difficulty to fit the varying cognitive abilities of neurotypical children and the different levels of impairment in children with ADHD, and to test the required amount of training to produce meaningful effects (i.e., the appropriate “dose” in pharmacological terms).

Another limitation is due to the pre-post study design, making it subject to the influence of confounding variables. This methodology was chosen because the digital therapy being tested is considered innovative, incorporating novel gamification elements such as reward schemes and storylines. Therefore, future research should employ a randomized controlled trial (RCT) design to more robustly support the effectiveness of this game in treating specific ADHD symptoms in Chinese children. Moreover, longitudinal studies based on this game’s design and its iterations are recommended to assess its long-term effectiveness. As the current study also did not make detailed comparisons among different ADHD subtypes, future studies should consider a more detailed grouping of ADHD patients.

Despite these limitations, the current study can be seen as an extensive conceptual examination of a novel serious game intervention aimed at reducing ADHD clinical symptoms from a clinical perspective. By setting improvements in social function as the training goal, this study’s findings support that this serious video game has demonstrated promising therapeutic effects. Although drugs remain the primary treatment for ADHD patients, the advent of the digital age promises more convenient, effective, and cost-efficient medical treatment, potentially benefiting a larger patient population [54].

## Conclusion

The findings advocate for the integration of serious video games as a complementary tool in ADHD treatment strategies, demonstrating the potential to augment attentional abilities and alleviate clinical symptoms. However, a randomized controlled trial (RCT) is needed to further verify its efficacy.

### Supplementary information

supplementary table 1

supplementary table 2

## Data Availability

The data that support the findings of this study are available on request from the corresponding author. The data are not publicly available due to privacy or ethical restrictions.

## References

[1] Li F, Cui Y, Li Y, Guo L, Ke X, Liu J (2022). Prevalence of mental disorders in school children and adolescents in China: diagnostic data from detailed clinical assessments of 17,524 individuals. J Child Psychol Psychiatry.

[2] Posner J, Polanczyk GV, Sonuga-Barke E (2020). Attention-deficit hyperactivity disorder. Lancet (London, England).

[3] Drechsler R, Brem S, Brandeis D, Grünblatt E, Berger G, Walitza S (2020). ADHD: current concepts and treatments in children and adolescents. Neuropediatrics.

[4] Anbarasan D, Kitchin M, Adler LA (2020). Screening for adult ADHD. Curr Psychiat Rep.

[5] Caye A, Swanson JM, Coghill D, Rohde LA (2019). Treatment strategies for ADHD: an evidence-based guide to select optimal treatment. Mol Psychiatry.

[6] Marcus SC, Durkin M (2011). Stimulant adherence and academic performance in urban youth with attention-deficit/hyperactivity disorder. J Am Acad Child Adolesc Psychiatry.

[7] Toomey SL, Sox CM, Rusinak D, Finkelstein JA (2012). Why do children with ADHD discontinue their medication?. Clin Pediatrics.

[8] Cortese S, Adamo N, Del Giovane C, Mohr-Jensen C, Hayes AJ, Carucci S (2018). Comparative efficacy and tolerability of medications for attention-deficit hyperactivity disorder in children, adolescents, and adults: a systematic review and network meta-analysis. Lancet. Psychiatry.

[9] Sayal K, Prasad V, Daley D, Ford T, Coghill D (2018). ADHD in children and young people: prevalence, care pathways, and service provision. Lancet. Psychiatry.

[10] He F, Qi Y, Zhou Y, Cao A, Yue X, Fang S (2023). Meta-analysis of the efficacy of digital therapies in children with attention-deficit hyperactivity disorder. Front Psychiatry.

[11] Krath J, Schürmann L, Von Korflesch HFO (2021). Revealing the theoretical basis of gamification: a systematic review and analysis of theory in research on gamification, serious games and game-based learning. Comput Hum Behav.

[12] Fleming TM, Cheek C, Merry SN, Thabrew H, Bridgman H, Stasiak K (2014). Serious games for the treatment or prevention of depression: a systematic review. Revista Psicopatología Psicología Clínica.

[13] Chaarani B, Ortigara J, Yuan D, Loso H, Potter A, Garavan HP (2022). Association of video gaming with cognitive performance among children. JAMA Netw Open.

[14] Anguera JA, Boccanfuso J, Rintoul JL, Al-Hashimi O, Faraji F, Janowich J (2013). Video game training enhances cognitive control in older adults. Nature.

[15] Kollins SH, Childress A, Heusser AC, Lutz J (2021). Effectiveness of a digital therapeutic as adjunct to treatment with medication in pediatric ADHD. Npj Digit Med.

[16] Kollins SH, DeLoss DJ, Cañadas E, Lutz J, Findling RL, Keefe RSE (2020). A novel digital intervention for actively reducing severity of paediatric ADHD (STARS-ADHD): a randomised controlled trial. Lancet Digital Health.

[17] Ismail FY, Fatemi A, Johnston MV (2017). Cerebral plasticity: windows of opportunity in the developing brain. Eur J Paediatric Neurol.

[18] Meyer KN, Santillana R, Miller B, Clapp W, Way M, Bridgman-Goines K (2020). Computer-based inhibitory control training in children with Attention-Deficit/Hyperactivity Disorder (ADHD): Evidence for behavioral and neural impact. PLoS ONE.

[19] Klingberg T, Fernell E, Olesen PJ, Johnson M, Gustafsson P, Dahlström K (2005). Computerized training of working memory in children with ADHD-a randomized, controlled trial. J Am Acad Child Adolescent Psychiatry.

[20] Bul KC, Franken IH, Van der Oord S, Kato PM, Danckaerts M, Vreeke LJ (2015). Development and user satisfaction of “Plan-It Commander,” a serious game for children with ADHD. Games Health J.

[21] Johnstone SJ, Roodenrys S, Blackman R, Johnston E, Loveday K, Mantz S (2012). Neurocognitive training for children with and without AD/HD. Atten Defic Hyperact Disord.

[22] Chacko A, Feirsen N, Bedard A, Marks D, Uderman JZ, Chimiklis A (2013). Cogmed working memory training for youth with ADHD: a closer examination of efficacy utilizing evidence-based criteria. J Clin Child Adolescent Psychol.

[23] Chacko A, Bedard AC, Marks DJ, Feirsen N, Uderman JZ, Chimiklis A (2014). A randomized clinical trial of Cogmed working memory training in school‐age children with ADHD: A replication in a diverse sample using a control condition. J Child Psychol Psychiatry.

[24] Westwood SJ, Parlatini V, Rubia K, Cortese S, Sonuga-Barke EJ (2023). Computerized cognitive training in attention-deficit/hyperactivity disorder (ADHD): a meta-analysis of randomized controlled trials with blinded and objective outcomes. Mol Psychiatry.

[25] Sújar A, Martín-Moratinos M, Rodrigo-Yanguas M, Bella-Fernández M, González-Tardón C, Delgado-Gómez D (2022). Developing serious video games to treat attention deficit hyperactivity disorder: tutorial guide. JMIR Serious Games.

[26] Sala G, Aksayli ND, Tatlidil KS, Tatsumi T, Gondo Y, Gobet F (2019). Near and far transfer in cognitive training: a second-order meta-analysis. Collabra: Psychol.

[27] Peñuelas-Calvo I, Jiang-Lin LK, Girela-Serrano B, Delgado-Gomez D, Navarro-Jimenez R, Baca-Garcia E (2022). Video games for the assessment and treatment of attention-deficit/hyperactivity disorder: a systematic review. Eur Child Adolesc Psychiatry.

[28] Furukawa E, Alsop B, Alves H, Vorderstrasse V, Carrasco KD, Chuang CC (2023). Disrupted waiting behavior in ADHD: exploring the impact of reward availability and predictive cues. Child Neuropsychol.

[29] Vajawat B, Varshney P, Banerjee D (2021). Digital gaming interventions in psychiatry: evidence, applications and challenges. Psychiat Res.

[30] First MB. DSM-5® handbook of differential diagnosis. American Psychiatric Pub; 2013.

[31] Wang Y, Zuo C, Xu Q, Hao L, Zhang Y (2021). Attention-deficit/hyperactivity disorder is characterized by a delay in subcortical maturation. Prog Neuro-Psychopharmacol Biol Psychiatry.

[32] Ramos AA, Hamdan AC, Machado L (2020). A meta-analysis on verbal working memory in children and adolescents with ADHD. Clin Neuropsychologist.

[33] Chen S, Yu J, Zhang Q, Zhang J, Zhang Y, Wang J (2022). Which factor is more relevant to the effectiveness of the cognitive intervention? A meta-analysis of randomized controlled trials of cognitive training on symptoms and executive function behaviors of children with attention deficit hyperactivity disorder. Front Psychol.

[34] Hall CL, Guo B, Valentine AZ, Groom MJ, Daley D, Sayal K (2020). The validity of the SNAP-IV in children displaying ADHD symptoms. Assessment.

[35] Goodman R (1997). The strengths and difficulties questionnaire: a research note. J Child Psychol Psychiatry.

[36] Gioia G, Isquith P, Guy SC, Kenworthy L (2000). Behavior rating inventory of executive function. Child Neuropsychol.

[37] Mirsky AF (1963). The Continuous Performance Test. The Genain quadruplets: a case study and theoretical analysis of heredity and environment in schizophrenia.

[38] Conners C, Epstein J, Angold A, Klaric J (2003). Continuous performance test performance in a normative epidemiological sample. J Abnorm Child Psychol.

[39] Van der Stigchel S, Meeter M, Theeuwes J (2006). Eye movement trajectories and what they tell us. Neurosci Biobehav R.

[40] Król ME, Król M (2020). The right look for the job: decoding cognitive processes involved in the task from spatial eye-movement patterns. Psychol Res.

[41] Coe BC, Munoz DP (2017). Mechanisms of saccade suppression revealed in the anti-saccade task. Phil Trans Roy Soc Lond Ser B: Biol Sci.

[42] Raabe M, Fischer V, Bernhardt D, Greenlee MW (2013). Neural correlates of spatial working memory load in a delayed match-to-sample saccade task. Neuroimage.

[43] Myers K, Winters NC (2002). Ten-year review of rating scales. I: overview of scale functioning, psychometric properties, and selection. J Am Acad Child Adolesc Psychiatry.

[44] Strahler Rivero T, Herrera Nuñez LM, Uehara Pires E, Amodeo Bueno OF (2015). ADHD rehabilitation through video gaming: a systematic review using PRISMA guidelines of the current findings and the associated risk of bias. Front Psychiatry.

[45] de Weijer AD, Mandl RC, Sommer IE, Vink M, Kahn RS, Neggers SF (2010). Human fronto-tectal and fronto-striatal-tectal pathways activate differently during anti-saccades. Front Hum Neurosci.

[46] Yep R, Smorenburg ML, Riek HC, Calancie OG, Kirkpatrick RH, Perkins JE (2022). Interleaved pro/anti-saccade behavior across the lifespan. Front Aging Neurosci.

[47] Klein C, Rauh R, Biscaldi M (2010). Cognitive correlates of anti-saccade task performance. Exp Brain Res.

[48] Irwin Harper LN, Groves NB, Marsh CL, Cole AM, Kofler MJ (2023). [Formula: see text] Does training working memory or inhibitory control produce far-transfer improvements in set shifting for children with ADHD? A randomized controlled trial. Child Neuropsychol.

[49] Levy F, Farrow M (2001). Working memory in ADHD: prefrontal/parietal connections. Curr Drug Targets.

[50] Hou L, Yang J, Xu L, Peng J, Joyce Law CY, Chen T (2023). Activation of brain regions associated with working memory and inhibitory control in patients with attention-deficit/hyperactivity disorder in functional near-infrared spectroscopy: a systematic review. Curr Med Imaging.

[51] Lee D (2013). Decision making: from neuroscience to psychiatry. Neuron.

[52] Amiri S, Shafiee-Kandjani AR, Noorazar SG, Rahmani Ivrigh S, Abdi S (2016). Knowledge and attitude of parents of children with attention deficit hyperactivity disorder towards the illness. Iran J Psychiatry Behav Sci.

[53] De Vet E, Simons M, Wesselman M (2014). Dutch children and parents’ views on active and non-active video gaming. Health Promot Int.

[54] Rodrigo-Yanguas M, González-Tardón C, Bella-Fernández M, Blasco-Fontecilla H (2022). Serious video games: angels or demons in patients with attention-deficit hyperactivity disorder? a quasi-systematic review. Front Psychiatry.

